# Social complexity parallels vocal complexity: a comparison of three non-human primate species

**DOI:** 10.3389/fpsyg.2013.00390

**Published:** 2013-07-09

**Authors:** Hélène Bouchet, Catherine Blois-Heulin, Alban Lemasson

**Affiliations:** ^1^Station Biologique, Laboratoire d'Éthologie Animale et Humaine EthoS – UMR 6552, Centre National de la Recherche Scientifique, Université de Rennes 1Paimpont, France; ^2^Primate Research Institute (Cognition and Learning section), Kyoto UniversityInuyama, Japan; ^3^Institut Universitaire de FranceParis, France

**Keywords:** evolution of communication, acoustic variability, acoustic individual distinctiveness, vocal repertoire, social system, Cercopithecus sp., Cercocebus sp.

## Abstract

Social factors play a key role in the structuring of vocal repertoires at the individual level, notably in non-human primates. Some authors suggested that, at the species level too, social life may have driven the evolution of communicative complexity, but this has rarely been empirically tested. Here, we use a comparative approach to address this issue. We investigated vocal variability, at both the call type and the repertoire levels, in three forest-dwelling species of *Cercopithecinae* presenting striking differences in their social systems, in terms of social organization as well as social structure. We collected female call recordings from twelve De Brazza's monkeys (*Cercopithecus neglectus*), six Campbell's monkeys (*Cercopithecus campbelli*) and seven red-capped mangabeys (*Cercocebus torquatus*) housed in similar conditions. First, we noted that the level of acoustic variability and individual distinctiveness found in several call types was related to their importance in social functioning. Contact calls, essential to intra-group cohesion, were the most individually distinctive regardless of the species, while threat calls were more structurally variable in mangabeys, the most “despotic” of our three species. Second, we found a parallel between the degree of complexity of the species' social structure and the size, diversity, and usage of its vocal repertoire. Mangabeys (most complex social structure) called twice as often as guenons and displayed the largest and most complex repertoire. De Brazza's monkeys (simplest social structure) displayed the smallest and simplest repertoire. Campbell's monkeys displayed an intermediate pattern. Providing evidence of higher levels of vocal variability in species presenting a more complex social system, our results are in line with the theory of a social-vocal coevolution of communicative abilities, opening new perspectives for comparative research on the evolution of communication systems in different animal taxa.

## Introduction

Identifying the key determining factors guiding the evolution of communication systems in animals is still a matter of strong debate in the scientific community. The evolution of vocal communication has often been said to be inseparable from the evolution of social life (Marler, 1977, p. 46; Waser, 1982, p. 118; Snowdon and Hausberger, 1997), notably in non-human primates (Lemasson, 2011), but this evolutionary hypothesis is hard to test empirically. A means of testing it is to investigate to what extent social complexity has played a role in the structuring of a species' vocal repertoire, such as by comparing species that differ in their social system (Lemasson, 2011; Freeberg et al., 2012).

Animals have traditionally been categorised as either vocal learners, for those who need social tutor models to learn the structure of vocalizations from (e.g., oscines, cetaceans, humans), or non-vocal learners, for those whose vocal development is strongly genetically determined (e.g., non-human primates). Whereas the former present an extensible vocal repertoire (e.g., new complex song notes can be socially learned every year in certain songbirds: Hausberger, 1997), the latter present a comparatively limited number of fixed call types. However, within fixed call types, refined acoustic changes can still be found (Hammerschmidt and Fischer, 2008). Interestingly, a growing number of studies have highlighted that, within species, social factors play a key role in the structuring of individual vocal repertoires in terms of both production and usage, even in the so-called non-vocal learners. First, comparisons of different conspecific social groups showed that group membership can be encoded in the acoustic structure of vocalizations (e.g., primates: Crockford et al., 2004; Tanaka et al., 2006; bats: Boughman and Wilkinson, 1998; birds: Hopp et al., 2001; Wright and Wilkinson, 2001). Second, within a social group, inter-individual comparisons revealed that social bonding was reflected in the structure or usage of vocalizations. (e.g., primates: Lemasson and Hausberger, 2004; Lemasson et al., 2011a; cetaceans: Smolker and Pepper, 1999; birds: Hausberger et al., 1995; Brown and Farabaugh, 1997), as was the hierarchical dominance rank (e.g., primates: Mitani and Nishida, 1993; Fischer et al., 2004). Those vocalizations, functioning as “social badges,” are typically contact calls or long-distance calls whose role is to maintain intra-group cohesion. Third, at the intra-individual level, social learning of vocal usage rules and adult-like acoustic structures has been demonstrated (e.g., primates: Snowdon et al., 1997; Lemasson et al., 2011b; cetaceans: McCowan and Reiss, 1997; birds: Nottebohm, 1970). Still at the intra-individual level, the assumption has been made that the social function of a call type may influence its level of acoustic variability (“call social function” hypothesis). In fact, Snowdon et al. (1997; p. 236) and Griebel and Oller (2008, p. 25) suggested that higher levels of acoustic variability may have been selected for in affiliative calls which are essential to the intra-group social functioning, while alarm calls may be more conservative. The structural variability expected in affiliative calls would be a means of encoding information about the caller's identity (inter-individual variability) or the context of emission (intra-individual variability), for example. Accordingly, the few attempts that have been made to compare the level of acoustic variability of functionally different call types throughout a species' vocal repertoire suggest a greater potential for identity coding in contact calls, compared to alarm or distress calls for example, in a few primate (Rendall et al., 1998, 2009; Lemasson and Hausberger, 2011; Bouchet et al., 2012a) and bird (Charrier et al., 2001) species.

At the inter-specific level, social complexity may have favoured the evolution of vocal complexity, in terms of both production and usage. The structural complexity of a system is defined as some function of the number of different parts it has and the irregularity of their arrangement; thus, heterogeneous, elaborate, or patternless systems are complex (McShea, 1991). Also, social complexity refers to the number of interacting individuals (i.e., group size, social network size), the different types of individuals (i.e., different types of social roles), and the frequency, diversity, and distribution of the interactions among them. Vocal complexity relates to the size and diversity of the vocal repertoire, its organisational structure (e.g., association patterns between sound units into calls, and calls into sequences), the level of intra- and inter-individual variability of the signals, and also the frequency and contextual diversity of usage of those signals. The species' social structure (i.e., group size and composition) has been hypothesized to account for the level of complexity of the vocal repertoire displayed by group members, in terms of both size (Blumstein and Armitage, 1997; McComb and Semple, 2005; Knotková et al., 2009; Gustison et al., 2012) and diversity (Freeberg, 2006; Freeberg and Harvey, 2008) (“socially-driven repertoire complexity” hypothesis). In addition, it has been suggested that group size may also influence vocal activity (i.e., calling rates). When group size increases, the difficulty to perform “bodily grooming” would be compensated by “vocal grooming”; calling would thus serve the function of maintaining group cohesion (Dunbar, 1998; Griebel and Oller, 2008) (“vocal grooming” hypothesis). Nevertheless, to date, those evolutionary hypotheses have only been tested separately, through inter-species comparisons based on reviews of the existing literature (e.g., primates: Dunbar, 1998, 2012; McComb and Semple, 2005; rodents: Blumstein and Armitage, 1997; Pollard and Blumstein, 2012; cetaceans: May-Collado et al., 2007; birds: Krams et al., 2012) or through inter-group comparisons (groups varying in size) at the intra-specific level (e.g., Carolina chickadees, *Poecile carolinensis*: Freeberg, 2006; Freeberg and Harvey, 2008). Also, those hypotheses remain to be formally tested jointly through a comparative study of multiple species.

To investigate the link between social and vocal complexity, we selected species presenting striking differences in their social systems, but being closely-related and originating from a similar habitat, as both phylogeny (Gautier, 1988; Cap et al., 2008; Thinh et al., 2011) and habitat (Brown et al., 1995; Daniel and Blumstein, 1998) are other factors known to constrain the shaping of animal vocal repertoires. Also, in the present study, we investigated vocal variability in three closely-related non-human primate species, namely De Brazza's monkey (*Cercopithecus neglectus*), Campbell's monkey (*Cercopithecus campbelli*) and red-capped mangabey (*Cercocebus torquatus*). They originate from comparable African tropical forests, and were studied here in similar, controlled captive conditions. They present interesting differences in their social systems, in both social organization (discrete hierarchy in guenons vs. strong hierarchy in mangabeys, rare physical interactions in guenons vs. relatively frequent physical interactions in mangabeys) and social structure (small family groups for De Brazza's monkeys, medium-size harems for Campbell's monkeys, and large multi-male multi-female groups for red-capped mangabeys) (Gautier-Hion and Gautier, 1978; Mitani, 1989; Matthews and Matthews, 2002; Lemasson et al., 2006; Mwenja, 2006; Ouattara et al., 2009b; Dolado and Beltran, 2012). We decided to focus on females as we expected the influence of social factors on vocal variability to be more striking in this sex class whose role in mediating intra-group relationships is predominant (Rowell, 1988). Indeed, in all three species, sex differences in vocal as well as non-vocal behaviour are sizeable, especially in guenons. Females form the group “social core”, they often interact socially and are very active vocally, whereas males are more peripheral both socially and spatially, they act mostly as group protectors and seldom vocalize (Gautier-Hion and Gautier, 1978; Oswald and Lockard, 1980; Rowell, 1988; Lemasson et al., 2006). Also, females frequently exchange contact calls with social partners, whereas males rather produce calls in response to external stimuli, such as a spotted danger (e.g., predator, tree fall, neighboring group) (Lemasson and Hausberger, 2004; Ouattara et al., 2009b; Bouchet et al., 2010, 2012b). Hence, we believe that if social factors guide vocal complexity in these species, females should be first impacted.

We evaluated vocal variability at both the call type and the repertoire levels across our three species. First, we tested whether or not the species' social organization affects the relationship between the social function of a given call type and its level of acoustic variability, estimated here in terms of intra-individual structural variability and individual acoustic distinctiveness. We compared three functionally equivalent call types (contact, threat and alarm calls) in the three species through measurements of the same set of temporal and frequency parameters. Calls essential to the intra-group social functioning that is contact calls in all species, but also threat calls in more “despotic” species, were expected to display higher levels of both intra- and inter-individual acoustic variability than alarm calls (“call social function” hypothesis). Second, we tested whether or not the species' social structure influences its level of vocal activity, as well as the size and diversity of its vocal repertoire. Species living in larger groups were predicted to vocalize more frequently (“vocal grooming” hypothesis) and to display a structurally larger and richer vocal repertoire (“socially-driven repertoire complexity” hypothesis).

## Materials and methods

### Species features

De Brazza's monkeys (*Cercopithecus neglectus*), Campbell's monkeys (*Cercopithecus campbelli*) and red-capped mangabeys (*Cercocebus torquatus*) are closely-related (*Cercopithecidae* family, *Cercopithecinae* subfamily: Grubb et al., 2003) and they originate from comparable African tropical forests (Gautier-Hion et al., 1999; Matthews and Matthews, 2002; Buzzard, 2006). However, they differ strikingly when it comes to their social structure. De Brazza's monkey live in small family groups (including generally one but sometimes up to three adult females), average group size ranging from three to seven individuals in the literature (Quris, 1976; Gautier-Hion and Gautier, 1978; Brennan, 1985; Decker, 1995; Mugambi et al., 1997; Mwenja, 2006; King, 2008). Campbell's monkeys live in harem groups (including three to eight adult females) whose average size ranges from seven to 13 individuals (Harding, 1984; Galat and Galat-Luong, 1985; Buzzard, 2006; Ouattara et al., 2009b). Red-capped mangabeys live in relatively large multi-male multi-female groups that often split into smaller foraging groups whose average group size ranges from 19 to 21 (Mitani, 1989; Matthews and Matthews, 2002). In the three species, females are the “social core” of the group, whereas males act mostly as group protectors (Gautier-Hion and Gautier, 1978; Oswald and Lockard, 1980; Rowell, 1988; Lemasson et al., 2006; Ouattara et al., 2009b). The two guenons also differ from the mangabey species regarding social organization. Like most forest guenons, De Brazza's and Campbell's monkeys' social organization is based on rare physical interactions and a discrete hierarchy (Gautier-Hion and Gautier, 1978; Rowell, 1988; Lemasson et al., 2006). Conversely, the social organization of red-capped mangabeys, like most baboons and macaques, is based on relatively frequent peaceful and agonistic interactions and a strong hierarchy (Rowell, 1988; Dolado and Beltran, 2012).

### Subjects and housing conditions

Call recordings were conducted with five groups of De Brazza's monkeys, one group of Campbell's monkeys and three groups of red-capped mangabeys housed in indoor-outdoor enclosures enriched with either perches, ropes, shrubs or trees, at the Station Biologique de Paimpont in France, and at Howletts and Port Lympne Wild Animal Parks in the UK (Table 1—for more details on the housing conditions, see Bouchet et al., 2010, 2012b; Lemasson and Hausberger, 2011). Cumulatively, we collected data from twelve De Brazza's monkey, six Campbell's monkey, and seven red-capped mangabey adult females.

**Table 1:** **Subjects' characteristics and study conditions**.

**Species**	**Group composition (♀: ♂: juveniles) and location**	**Females' age when studied (in years)**	**Study period**
De Brazza's monkeys	1: 3: 3	3/6/19	August–September 2007
	Station Biologique de Paimpont		
	1: 1: 1	12	August–September 2007
	Station Biologique de Paimpont		
	1: 2: 2	3/16	October 2008
	Howletts Wild Animal Park		
	1: 4: 1	3/5/9/22	November 2008
	Port Lympne Wild Animal Park		
	1: 2: 0	3/15	November 2008
	Port Lympne Wild Animal Park^a^		
Campbell's monkeys	1: 8: 2	matriline #1: 4/5/8, matriline #2: 3/4/7	September–October 2000
	Station Biologique de Paimpont^b^		
Red-capped mangabeys	1: 1: 0	26	February–April 2008
	Station Biologique de Paimpont		
	0: 3: 2	6/8/21	February–April 2008
	Station Biologique de Paimpont		
	1: 3: 3	matriline #1: 4/16, matriline #2: 12	February–April 2008
	Station Biologique de Paimpont		

We considered females as sexually mature when more than 3 years old in De Brazza's and Campbell's monkeys (Gautier-Hion and Gautier, 1978; Galat and Galat-Luong, 1985), and when more than 4 years old in red-capped mangabeys (Hill, 1974).

a In this group, observations (8 h15 in total) were conducted ad-libitum, thus those data have been used in structural analysis, but not for call rate analysis.

b Two adult females were carrying an infant, and in order to avoid any disturbance, they were not equipped with a telemetric harness (Lemasson and Hausberger, 2011).

Subjects were provided with fruit, vegetables, and commercial monkey chow daily. Water was available *ad-libitum*. Animal care and research protocols used in this work complied with the current laws of France and the UK and were conducted under license from the Direction Départementale des Services Vétérinaires (DDSV n° 04672).

### Data collection

Observations were performed outdoors (to ensure high quality recordings suitable for acoustic analysis) between 09:00 and 18:00, outside of feeding times. Subjects were observed in a random order using the focal sampling method, sessions being distributed throughout the day for every subject. In De Brazza's monkeys and red-capped mangabeys, calls of focal (and occasionally of non-focal) individuals were recorded using a directional microphone (Sony ECM-672 for the females housed in Paimpont, Sennheiser MKH-70 for the females housed in Howletts and Port Lympne) connected to a digital stereo recorder (Sony DAT TCD-D100 for the De Brazza's monkeys housed in Paimpont, Marantz PMD-660 for the other groups). In guenons, females frequently produce soft sounds with their mouths closed, which made it difficult to identify the caller within a multi-female group, even in captivity. Thus, sound recordings in Campbell's monkeys were performed through a telemetric system composed of a transmitter (fixed on a leather harness on the monkey), a receiver, and a digital stereo recorder (TASCAM DA-P1) (for technical details, see Lemasson and Hausberger, 2011).

In total, 37 h50 of focal observations (3 h47 ± 1 h23 per individual, *N* = 10) were made on De Brazza's monkeys (plus 8 h15 *ad-libitum* on two adult females at Port Lympne, see Table 1), 92 h20 of telemetric recordings (15 h11 ± 4 h10 per individual, *N* = 6) were performed with Campbell's monkeys, and 34 h00 of focal observations (4 h50 ± 0 h16 per individual, *N* = 7) were conducted on red-capped mangabeys. We were able to extract from these recordings 1569 De Brazza's monkey calls (66–336 per individual), 1309 Campbell's monkey calls (99–466 per individual) and 3970 red-capped mangabey calls (163–939 per individual).

### Data analysis

#### Call types analysis

**Figure 1:**
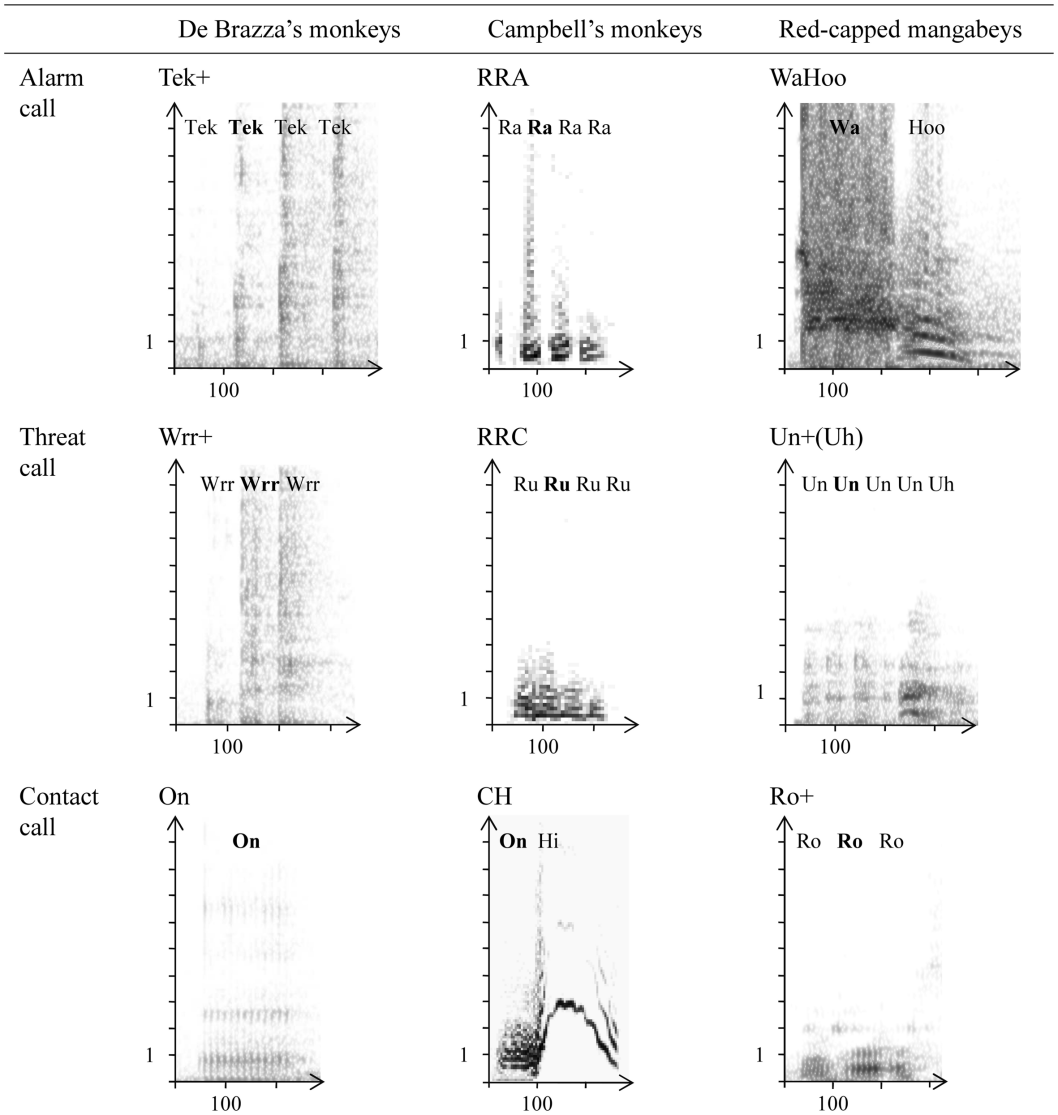
**Sonograms of contact, threat and alarm calls in De Brazza's monkeys, Campbell's monkeys, and red-capped mangabeys**. Sonograms: X-axis: duration (ms), Y-axis: frequency (kHz). The call type name is given above the sonogram call types are labeled as in previous publications (De Brazza's monkeys: Bouchet et al., 2012b; Campbell's monkeys: Lemasson and Hausberger, 2011; red-capped mangabeys: Bouchet et al., 2010). Each sonogram is labeled according to the composition in units of the call (units are named using onomatopoeia), and the unit on which measurements have been performed is set in bold type. In the three species, alarm calls display a noisy pattern. In guenons, alarm calls (“Tek+” and “RRA”) consist of repeated pulses, while red-capped mangabey alarm calls (“WaHoo”) are systematically composed of two units of different types. Guenon threat calls (“Wrr+” and “RRC”) display an acoustic pattern similar to that of their alarm calls, whereas red-capped mangabey threat calls (“Un+(Uh)”) are composed of one or several short low-pitched tonal units, sometimes combined to an additional “Uh” unit. In the three species, contact calls are composed of one short, low-pitched, tonal unit which is uttered mostly singularly in De Brazza's monkeys (“On”), combined to a second arched-shape high-pitched unit in Campbell's monkeys (“CH”), and either singularly or repeated in red-capped mangabeys (“Ro+”).

The vocal repertoires of the three species based on acoustic, contextual, and phylogenetic analyses have been described earlier (guenons: Gautier, 1988; De Brazza's monkeys: Bouchet et al., 2012b; Campbell's monkeys: Lemasson and Hausberger, 2011; red-capped mangabeys: Bouchet et al., 2010). We focused on three call types that are contextually equivalent across the three species but differ in their social function: a contact call (produced mostly during affiliative interactions), a threat call (uttered by aggressors during agonistic interactions) and an alarm call (elicited by external disturbances) (Figure 1).

Sonograms were drawn by a Fast-Fourier Transform (1024-pt FFT length containing 256 samples and zero padding, incremental step: 32 pt) using ANA acoustic software, a customized AMIGA micro-computer program (Richard, 1991) recently adapted to be run on PC implemented on LINUX. Calls recorded during both focal and non-focal observations were examined here, but signals with excessive background noise were excluded from the analyses. In order to best control for data set balance, we randomly selected, when possible, ten exemplars per call type and per individual (sample sizes are given in Table 2). We performed measurements at both the sound unit and the call levels (time resolution: 2.49 ms, frequency resolution: 50 Hz). A unit was defined as the basic element of a call, represented as a continuous tracing along the temporal axis of the sonogram (also referred to as “note” or “syllable” in the monkeys and birds literature, see Bouchet et al., 2010). For calls composed of more than one unit type (“CH”, “WaHoo”, “Un+(Uh)”), we performed measurements on the “principal unit” type only (i.e., the unit type introducing the call, as defined in Bouchet et al., 2010) (respectively, “On,” “Wa,” and “Un”) (Figure 1). Also, if a call consisted of several same-type units (“Tek+,” “Wrr+,” “RRA,” “RRC,” “Ro+,” “Un+(Uh)”), we performed measurements on the second unit in the call because the first unit was often not clearly visible (Figure 1). As we faced both noisy and tonal calls, we focused on five acoustic parameters that were shared by all call types: call duration (Dcall, ms), number of units (#units), unit duration (Dunit, ms), unit base frequency (Fbase, Hz; i.e., lowest-pitched reinforced frequency measured in the middle of the frequency band that is the fundamental frequency in tonal calls), and unit peak frequency (Fpeak, Hz; i.e., frequency at maximum energy, measured on the power spectrum) (following the same methodology as Bouchet et al., 2010, 2012a). In order to describe call types, as individuals contributed differently to the data set, we first calculated individual means and then averaged these scores. Standard deviations (SD) were calculated by averaging the data set SD of every individual and totaling it with the SD of the individual means (Table 2).

**Table 2:** **Call type characteristics and sample sizes**.

**Species**		**De Brazza's monkeys**			**Campbell's monkeys**			**Red-capped mangabeys**		
**Call type name^a^**		**Tek+**	**Wrr+**	**On**	**RRA**	**RRC**	**CH**	**WaHoo**	**Un+(Uh)**	**Ro+**
**Call function**		**Alarm**	**Threat**	**Contact**	**Alarm**	**Threat**	**Contact**	**Alarm**	**Threat**	**Contact**
	*N*_ind_	9	7	12	6	2	6	2	5	7
	*N*_calls_	52	79	120	54	14	60	15	38	70
Dcall (ms)	$\bar{X}\pm SDintra$	211 ± 112	218 ± 116	209 ± 125	132 ± 43	97 ± 38	264 ± 139	400 ± 95	289 ± 200	169 ± 101
#units	$\bar{X}\pm SDintra$	3 ± 1	3 ± 2	1 ± 0	3 ± 1	3 ± 2	2 ± 0	2 ± 0	4 ± 2	2 ± 1
Dunit (ms)	$\bar{X}\pm SDintra$	49 ± 17	55 ± 16	208 ± 125	27 ± 8	35 ± 14	105 ± 48	218 ± 84	63 ± 18	62 ± 29
Fbase (Hz)	$\bar{X}\pm SDintra$	194 ± 66	172 ± 39	93 ± 21	213 ± 65	534 ± 41	308 ± 41	1353 ± 115	188 ± 52	211 ± 69
Fpeak (Hz)	$\bar{X}\pm SDintra$	2888 ± 1652	270 ± 287	489 ± 455	781 ± 571	547 ± 264	911 ± 311	1880 ± 525	618 ± 617	411 ± 79

N_ind_, number of individuals contributing to the dataset; N_calls_, number of samples measured; $\bar{X}\pm SDintra$, intra-individual mean and associated standard deviation for each parameter (see the Data Analysis section for definitions); Dcall, call duration (ms), #units, number of units; Dunit, unit duration (ms), Fbase, unit base frequency (Hz); and Fpeak, unit peak frequency (Hz).

a As labeled in previous publications (De Brazza's monkeys: Bouchet et al., 2012b; Campbell's monkeys: Lemasson and Hausberger, 2011; red-capped mangabeys: Bouchet et al., 2010).

#### Vocal activity analysis

For each individual, we calculated the global hourly call rate (all call types merged), by dividing the total number of calls given during focal samples by the sum of the total observation time (in hours) for that individual. Individual means were then averaged within each species.

#### Repertoire analysis

The structural acoustic complexity of each species' repertoire was assessed at the sound unit level, using vocalizations recorded during focal observations only (to control for relative proportions of each type within the entire repertoire). We noted whether each call within the entire repertoire was composed either of a single unit (“single”), of multiple units of the same type (“repeated”), or of units of different types (“combined”). We described all the “unit assembling patterns,” and estimated their frequency of occurrence in each individual, then averaged within each species. We also computed the frequency of occurrence for the categories “single,” “repeated,” and “combined” patterns (whatever the unit types) at the individual level, then the species level.

### Statistical analysis

#### Call types analysis

To describe the pattern variability of each call type, we used coefficient of variation measurements. For each of the five acoustic parameters, and for every call type in each species, we computed the coefficient of intra-individual variation (CV_intra_ = mean of individual CV values, with for each individual: $CV=100\times \text{SD}/\bar{X}$), and then averaged it over the five acoustic parameters within each call type to compute an overall value: the call type intra-individual CV (CVmean_intra_ = *CV*_intra_ averaged over the five parameters). Then, we performed comparisons between call types and between species. Within each species, we tested whether the three call types differed in their level of intra-individual variability by performing Friedman tests on the five CV_intra_ values. Likewise, for each call type, we tested whether the three species differed by performing Friedman tests on the five CV_intra_ values. In both cases, Friedman tests were followed by *post-hoc* multiple comparisons (Siegel and Castellan, 1988).

To quantify the potential of each call type to encode identity, we used information capacity measurements, as described in the information analysis method developed by Beecher (1989) based on information theory (Shannon and Weaver, 1949). This approach has been used successfully in recent studies of birds (Searby et al., 2004) and mammals (Sèbe et al., 2010; Pollard and Blumstein, 2011). For each of the five acoustic parameters, and for every call type in each species, a One-Way analysis of variance (ANOVA, type III) was conducted with “caller identity' as the fixed factor. For variables giving a significant *F* (*P* ≤ 0.05), we then computed the stereotypy index *Hs* (Searby et al., 2004; Pollard and Blumstein, 2011). The information content of one parameter, *Hs*, is derived from the *F*-value found in the One-Way ANOVA, but, unlike *F*, *Hs* does not vary with sample size. Information capacity measurements are therefore more reliable when comparing samples. For a given parameter, *Hs* is expressed as:

$$Hs=\log_{2}\text{​}\left(\sqrt{\frac{F\times n(k-1)}{k(n-k)}}\right)$$

where *F* = result of ANOVA, *n* = number of calls, and *k* = number of individuals contributing to the dataset. *Hs* (in bits/signal) represents the number of binary decisions necessary to discriminate between *N* objects. The higher the value of *Hs*, the greater potential the parameter has for encoding individual identity. Parameters with an *Hs* < 1 can be considered as “low-informative” (Searby et al., 2004). Then, we computed the total *Hs* information for each call type (Σ*Hs*) that is, the sum of the information in each of the independent variables. Since some of the acoustic parameters measured are likely to be inter-correlated, we combined the five variables into principal components (Principal Component Analysis with Varimax rotation), and retained those giving a significant *F* (*P* ≤ 0.05) to calculate the total *Hs* information (Beecher, 1989; Sèbe et al., 2010).

#### Vocal activity analysis

We compared vocal activity between the three species using a Kruskall-Wallis test on the global hourly call rates computed for every individual within each species. Then, we performed *post-hoc* one-tailed pairwise comparisons (Siegel and Castellan, 1988) chosen according to the “vocal grooming” hypothesis (H1: “De Brazza's monkeys” < “Campbell's monkeys”, H1: “De Brazza's monkeys” < “red-capped mangabeys”, and H1: “Campbell's monkeys” < “red-capped mangabeys”).

#### Repertoire analysis

To estimate the structural complexity of each species' vocal repertoire in terms of “unit assembling patterns”, we computed a diversity index following a procedure introduced by Shannon and Weaver (1949). This involves the calculation of two indices: *H*_*i*_ max represents the value if all signal types are uttered with the same frequency, while *H*_*i*_ represents the actual observed values. The index of diversity *DI* is thus expressed as:

$$DI=\frac{H_{i}}{H_{i}\max}=\frac{-\sum_{i=1}^{n}p_{i}\times \log_{2}(p_{i})}{\log_{2}(n)}$$

where *n* = total number of different “unit assembling pattern” types, and *p*_*i*_ = the probability of occurrence of each pattern. The smaller the value of *DI*, the less diverse the repertoire (i.e., the repertoire is dominated by one “unit assembling pattern” type).

We also compared the complexity of vocal production between the three species. We performed Kruskall-Wallis tests on the frequencies of utterance computed for the categories “single,” “repeated,” and “combined” patterns separately, followed by *post-hoc* multiple comparisons (Siegel and Castellan, 1988). Furthermore, within each species, we tested whether one category of pattern (“single,” “repeated,” or “combined”) was more frequent than another by performing Friedman tests followed by *post-hoc* multiple comparisons (Siegel and Castellan, 1988).

Statistical analyses were performed using SPSS 13.0, Minitab 12.2 and Microsoft Excel. We set significance at α = 0.05.

## Results

### Call types' level of variability and individual distinctiveness

**Figure 2:**
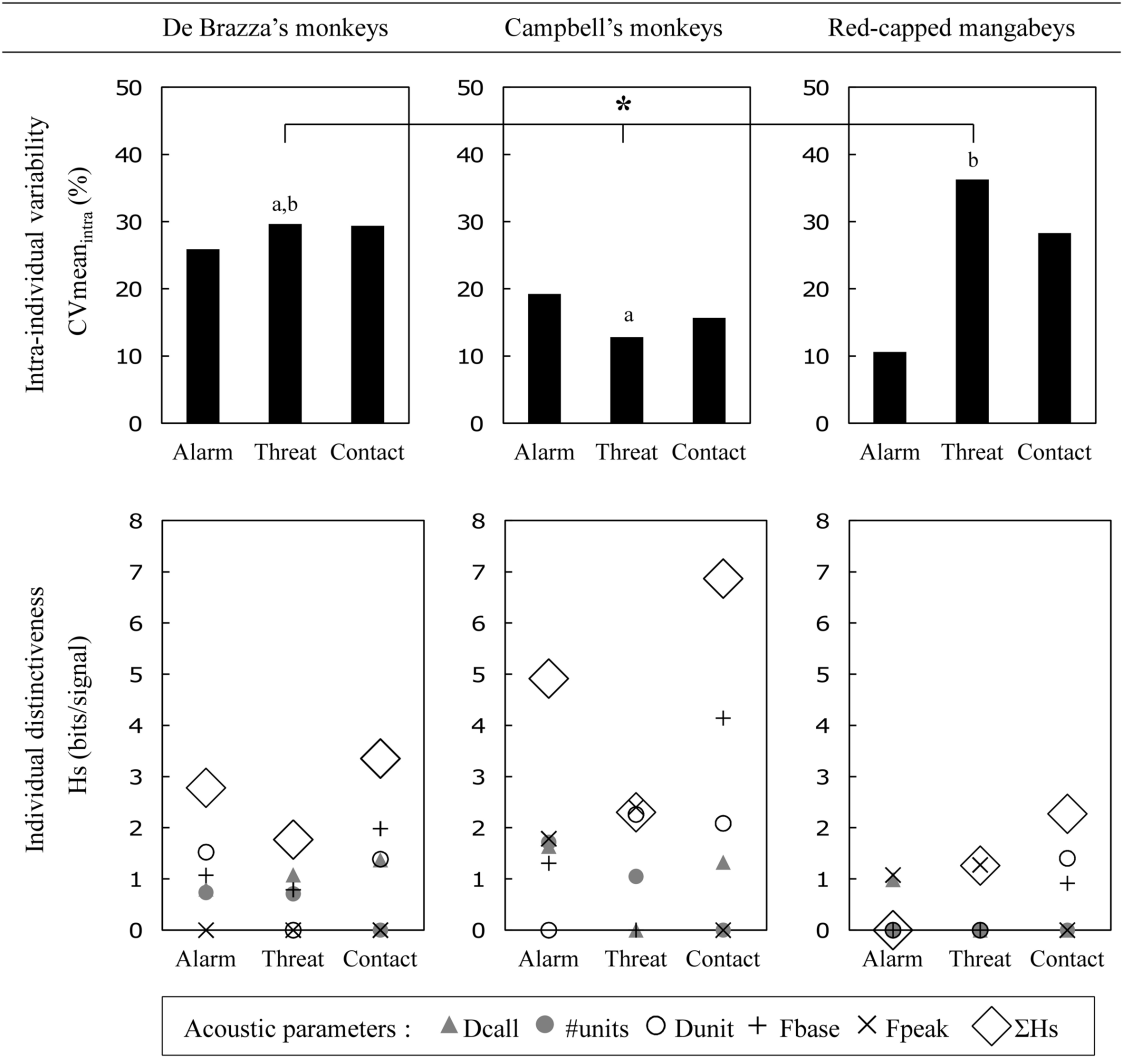
**Acoustic intra-individual variability and individual distinctiveness in contact, threat and alarm calls in each of three species of primate**. CVmean_intra_: call type intra-individual coefficient of variation (%) and Friedman test result (^*^P < 0.05; results of the *post-hoc* multiple comparisons are illustrated by letters symbolizing homogeneous subsets). *Hs*: information content value (see the Statistical Analysis section for definitions). Dcall, call duration (ms); #units, number of units; Dunit, unit duration (ms); Fbase, unit base frequency (Hz); and Fpeak, unit peak frequency (Hz).

The overall level of structural intra-individual variability as well as the level of individual distinctiveness varied among the three functionally different call types (contact, threat and alarm; Figure 1) across the three species (Figure 2).

The level of intra-individual variation in the call acoustic properties did not differ significantly between call types within a species' repertoire (Friedman tests: De Brazza's monkeys: χ^2^_2_ = 0, *P* = 1; Campbell's monkeys: χ^2^_2_ = 2.80, *P* = 0.247; red-capped mangabeys: χ^2^_2_ = 4.80, *P* = 0.091) (Figure 2). However, it significantly differed between species for the threat call; female red-capped mangabeys produced the most variable threat call pattern (Friedman test: χ^2^_2_ = 7.60, *P* = 0.022; notably because red-capped mangabey threat calls were significantly more variable than those of Campbell's monkeys, *post-hoc* multiple comparisons: *P* < 0.05) (Figure 2). No inter-specific differences were observed for the two other call types (Friedman tests: alarm calls: χ^2^_2_ = 3.60, *P* = 0.165; contact calls: χ^2^_2_ = 4.80, *P* = 0.091) (Figure 2).

Interestingly, the level of inter-individual variation did not correlate with the level of intra-individual variation found in the various call types. In the three species, the potential for identity coding was the highest in contact calls (highest Σ*Hs* values) (Figure 2). However, while red-capped mangabey threat calls displayed a higher potential for identity coding (higher Σ*Hs* value) than their alarm calls, the two guenon species displayed the opposite pattern (Figure 2). Regarding the ability to encode individual identity of the various acoustic parameters, it appeared that the number of units per call was low-informative (*Hs* < 1 in all cases but Campbell's monkeys' alarm calls) while the frequency parameters (base and/or peak frequencies) were informative regarding identity in most cases (one or both frequency parameters had a *Hs* > 1 in each call type, except threat calls in De Brazza's monkeys and contact calls in red-capped mangabeys) (Figure 2). Conversely to frequency parameters, no systematic pattern of variability was found across species for temporal features (call and unit duration), except that unit duration was informative (*Hs* > 1) for contact calls in the three species (Figure 2).

### Vocal activity across species

**Figure 3:**
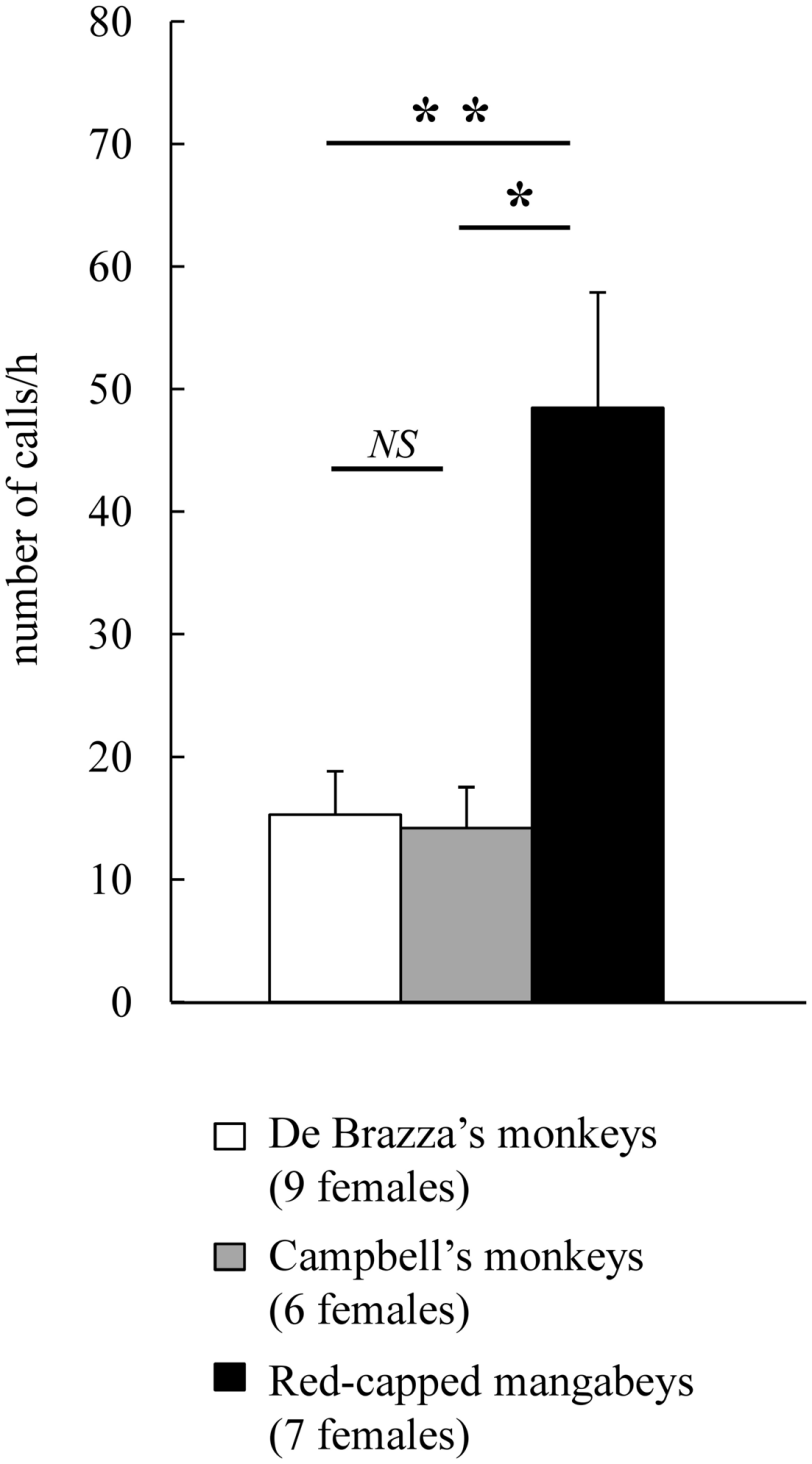
**Hourly call rates in each of three species of primate**. Number of calls/h: $\bar{X}\pm SE$. Results of the *post-hoc* multiple comparisons following the Kruskall-Wallis test performed after removing the outlier (one De Brazza's monkey female). The one-tailed pairwise comparisons were chosen according to the “vocal grooming” hypothesis (H1: “De Brazza's monkeys” < “Campbell's monkeys,” H1: “De Brazza's monkeys” < “red-capped mangabeys,” and H1: “Campbell's monkeys” < “red-capped mangabeys”) (^**^P < 0.025, ^*^P < 0.05, NS, not significant).

Red-capped mangabeys and the two guenon species differed in their global vocal activity, the former calling more than twice as much as the two others (Kruskall-Wallis test: *H*_2_ = 5.96, *P* = 0.051). The *post-hoc* one-tailed pairwise comparisons (chosen according to the “vocal grooming” hypothesis) revealed that call rates were significantly higher in red-capped mangabeys than Campbell's monkeys (*P* < 0.05), were higher in red-capped mangabeys than De Brazza's monkeys, although not significantly so (*P* < 0.075), and did not differ between the two guenon species (*P* > 0.15). However, we noted that one De Brazza's monkey female (102.5 calls/h) called ten times as much as the nine other conspecific females ($\bar{X}$ ± *SE*: 15.3 ± 3.6 calls/h; range: 4.8–38.5). Therefore, we excluded this outlier (Grubb's test: *Z* = 2.673, *N* = 10) and reran the analyses (Figure 3). The global test result did not change (Kruskall-Wallis test: *H*_2_ = 7.75, *P* = 0.021), but the *post-hoc* one-tailed pairwise comparisons revealed that while call rates remained significantly higher in red-capped mangabeys than Campbell's monkeys (*P* < 0.05), the difference between red-capped mangabeys and De Brazza's monkeys was now significant as well (*P* < 0.025). We still did not observe a difference between the two guenon species (*P* > 0.15).

In the three species, contact calls dominated the females' vocal repertoire, accounting for more than 60% of the total vocal production. Also, red-capped mangabeys produced more contact calls than the two guenon species ($\bar{X}$ ± *SE*: red-capped mangabeys “Ro+”: 29.8 ± 9.2 calls/h; Campbell's monkeys “CH”: 8.6 ± 2.8 calls/h; De Brazza's monkeys “On”: 19.4 ± 9.5 calls/h; De Brazza's monkeys “On” without the outlier: 10.4 ± 3.2 calls/h). In addition, red-capped mangabeys uttered threat calls more than three times as much as the two guenon species (red-capped mangabeys “Un+(Uh)”: 7.7 ± 4.5 calls/h; Campbell's monkeys “RRC”: 0.2 ± 0.1 calls/h; De Brazza's monkeys “Wrr+”: 2.4 ± 0.8 calls/h; De Brazza's monkeys “Wrr+” without the outlier: 2.5 ± 0.9 calls/h).

### Repertoire complexity across species

**Figure 4:**
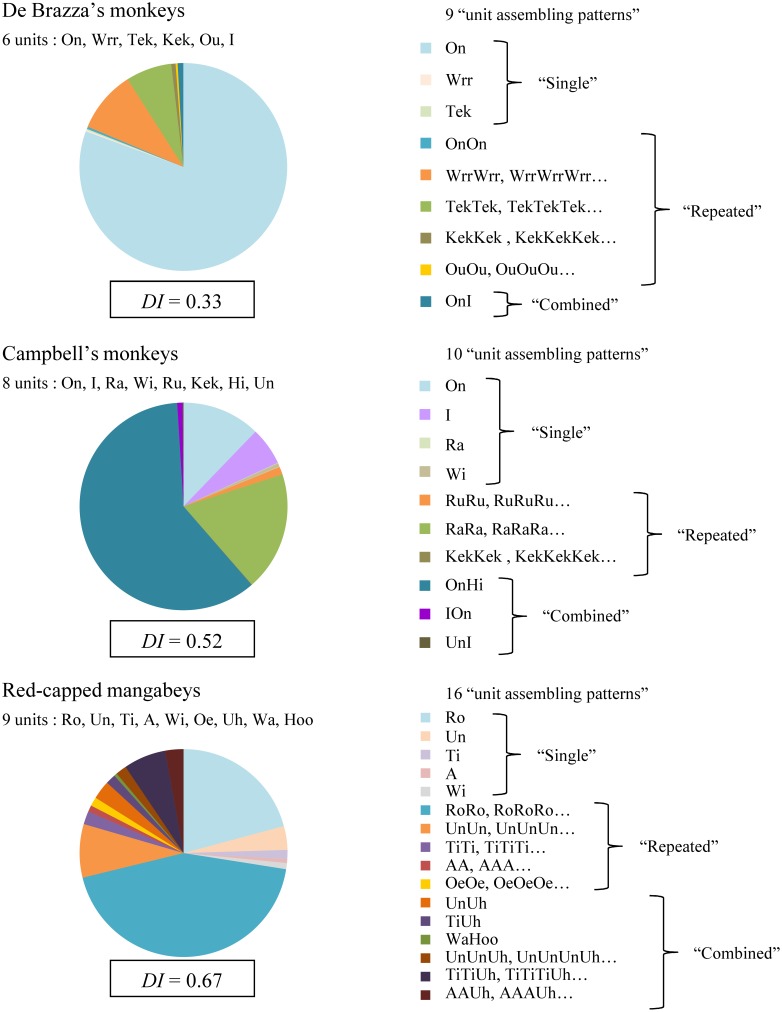
**Relative proportion of the different “unit assembling patterns” produced by each of three species of primate**. Below the species' name: number and name of unit types. Unit types are labeled as in previous publications for De Brazza's monkeys (Bouchet et al., 2012b) and red-capped mangabeys (Bouchet et al., 2010). Campbell's monkeys' repertoire, however, has been revised according to Bouchet et al. (2010) multi-level approach; the correspondence between this nomenclature and the call type names used in Lemasson and Hausberger (2011) is as follow: “On” = SH; “I” = ST; “Ra, RaRa, RaRaRa …” = RRA; “Wi” = SA; “RuRu, RuRuRu …” = RRC; “KekKek, KekKekKek …” = RSA; “OnHi” = CH; “IOn” = CT; “UnI” = RST. On the right-hand side: all the unit assembling patterns observed, grouped as “single”, “repeated”, “combined” (see the Data Analysis section for definitions). Below each diagram: *DI*, diversity index (Shannon and Weaver, 1949). The smaller the value of *DI*, the less diverse the repertoire.

When assessing the structural acoustic variability of each species' at the sound unit level, we identified 6 unit types in De Brazza's monkeys, 8 unit types in Campbell's monkeys, and 9 unit types in red-capped mangabeys (Figure 4). Those units were then concatenated into 9, 10 and 16 different “unit assembling patterns” respectively. Interestingly, the number of “combined” patterns was the lowest in De Brazza's monkeys (*N* = 1), intermediate in Campbell's monkeys (*N* = 3), and the highest in red-capped mangabeys (*N* = 6) (Figure 4).

The diversity index *DI* enabled us to estimate the structural complexity of each species' vocal repertoire in terms of “unit assembling pattern” types (Figure 4). As a result, it appeared that female De Brazza's monkeys displayed the less diverse repertoire (*DI* = 0.33), female red-capped mangabeys the most diverse (*DI* = 0.67), while female Campbell's monkeys showed an intermediate pattern (*DI* = 0.52). Indeed, the De Brazza's monkeys' repertoire was largely dominated by the contact call in its “single” form (80.6%), while that of Campbell's monkeys was dominated by the contact call in its “combined” pattern (60.8%). In contrast, the repertoire of red-capped mangabeys was dominated by the contact call in its “repeated” form (43.7%), followed by its “single” form (20.8%), but it was also largely composed of many different “unit assembling patterns” in various proportions (range: 0.5–8.3%) (Figure 4).

**Figure 5:**
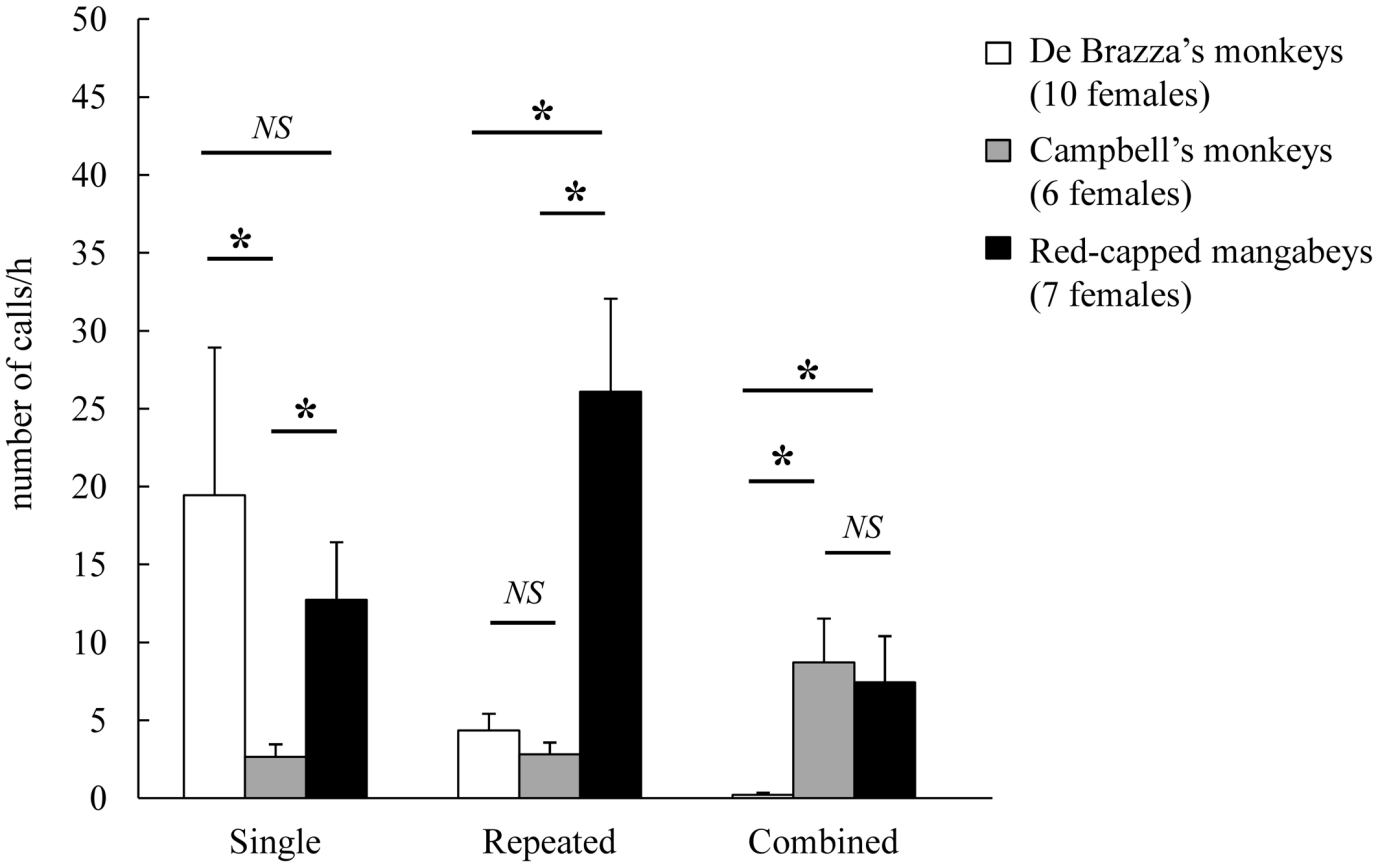
**Hourly call rate of “single”, “repeated” and “combined” unit assembling patterns in each of three species of primate**. Number of calls/h: $\bar{X}\pm SE$. Results of the *post-hoc* multiple comparisons following the Kruskall-Wallis test (^*^P < 0.05, NS, not significant).

When performing intra-specific comparisons regarding pattern categories (Figure 5), we found that De Brazza's monkeys produced mostly “single” patterns (81.0%; Friedman tests: χ^2^_2_ = 14.00, *P* = 0.001; notably because “single” patterns were more frequent than “combined” patterns, *post-hoc* multiple comparisons: *P* < 0.05), while red-capped mangabeys uttered mostly “repeated” patterns (56.4%; Friedman tests: χ^2^_2_ = 11.14, *P* = 0.004; “repeated” were more frequent than both “single” and “combined” patterns, *post-hoc* multiple comparisons: *P* < 0.05). No significant preference was found for Campbell's monkeys (Friedman tests: χ^2^_2_ = 4.00, *P* = 0.135) (Figure 5).

Furthermore, we found that “single” patterns were more frequently uttered by both De Brazza's monkeys and red-capped mangabeys than Campbell's monkeys (Kruskall-Wallis tests: *H*_2_ = 7.48, *P* = 0.024). “Repeated” patterns were above all produced by red-capped mangabeys (*H*_2_ = 13.02, *P* = 0.001). Lastly, “combined” patterns were more frequently uttered by both Campbell's monkeys and red-capped mangabeys (*H*_2_ = 16.03, *P* = 0.001) (see Figure 5 for the results of the *post-hoc* multiple comparisons).

## Discussion

Several findings emerge from this comparative study of the level of vocal variability in three closely related but socially distinct non-human primate species. Overall, we found evidence that the species' social structure and social organization covary with the level of complexity observed in its vocal repertoire, in terms of both structuring and usage; our results are in line with the predictions of the “social system—vocal variability” coevolution hypothesis. When comparing three functionally different call types (contact, threat and alarm), acoustic variability did not appear homogeneously within nor across species. Individual distinctiveness was the highest in contact calls regardless of the species examined. In contrast, threat calls were the least individually distinctive in the “tolerant' guenons, but displayed an intermediate pattern compared with contact and alarm calls in the “despotic” mangabeys. Meanwhile, female mangabey threat calls were more structurally variable than those of female guenons. These results support the “call social function” hypothesis. When investigating vocal activity, we found that calling rates of female mangabeys were more than twice as high as those of female guenons. Given that group size is larger in mangabeys compared with guenons in the wild, our result supports the “vocal grooming” hypothesis. Lastly, when investigating vocal repertoire structuring, we observed the largest and most diverse repertoire (i.e., more unit types, more assembling patterns, higher diversity index) in female red-capped mangabeys, the smallest and least diverse in female De Brazza's monkeys, and an intermediate pattern in Campbell's monkeys. All species enriched their repertoire diversity by producing their sound units singly, or in repeated or combined patterns. Interestingly, we found that the number of combinatorial patterns was the highest in female mangabeys, the lowest in De Brazza's monkeys, and intermediate in Campbell's monkeys. As repertoire complexity matches social structure complexity, these results support the “socially-driven repertoire complexity” hypothesis.

### Perceptual salience of the observed acoustic variability

It has to be acknowledged that the acoustic variability evidenced in this study, both at the call and the repertoire levels, remains to be tested for its relevance to listeners. Nonetheless, many studies have shown that non-human primates are able to discriminate between individuals from their calls (e.g., rhesus macaques, *Macaca mulatta*: Rendall et al., 1996; olive baboons, *Papio hamadryas anubis*: Lemasson et al., 2008; chacma baboons, *Papio hamadryas ursinus*: Rendall et al., 2009), can perceive subtle differences in acoustic structure (e.g., Campbell's monkeys: Lemasson et al., 2005), and can distinguish between sequences of sound units (e.g., cotton-top tamarins, *Saguinus oedipus*: Hauser et al., 2001; Diana monkeys, *Cercopithecus diana*: Zuberbühler, 2002; white-handed gibbons, *Hylobates lar*: Clarke et al., 2006; putty-nosed monkeys, *Cercopithecus nictitans*: Arnold and Zuberbühler, 2008; Campbell's monkeys: Lemasson et al., 2011a). Such evidence allows us to hypothesize that the acoustic variability observed here is perceptible to monkeys; future research must, however, determine its actual salience to listeners.

### Influence of social organization on call type design

In accordance with the “call social function” hypothesis (Snowdon et al., 1997; Griebel and Oller, 2008), we found the highest level of individual distinctiveness in contact calls, compared with alarm calls, in the three studied species. This is in agreement with the few studies in non-human primates and birds that have provided evidence of a higher level of individual distinctiveness in affiliative calls compared with distress, alarm, or courtship calls (Rendall et al., 1998, 2009; Charrier et al., 2001; Lemasson and Hausberger, 2011; Bouchet et al., 2012a). Interestingly, we provided further evidence of an effect of the social organization (in terms of strong vs. discrete hierarchy) on the level of acoustic variability of the threat call. In fact, we found a greater level of intra-individual structural variability in the threat calls of the “despotic” red-capped mangabeys than in those of the two “tolerant” guenon species. Moreover, we found that identity coding was stronger in female mangabey threat vs. alarm calls, whereas in guenons threat calls were the least individually distinctive. Consequently, we suggest that the call's relevance to the species' social organization (i.e., whether or not the call mediates intra-group interactions, which are essential to social functioning), rather than the type of message it conveys (e.g., contact vs. threat vs. alarm), accounts for its level of acoustic variability. Furthermore, we found that the degree of individual distinctiveness of a call type did not correlate with its level of intra-individual acoustic variability. Also, it seems that in the case of mangabey threat calls, the realized acoustic variability could serve to encode information not only about the caller's identity but also about the context of emission (e.g., rhesus macaques: Gouzoules et al., 1984; chimpanzees, *Pan troglodytes*: Slocombe and Zuberbühler, 2007).

Whereas contact calls and alarm calls displayed similar frequency patterns (tonal and noisy respectively) in the three species we studied, threat call structural patterns differed across species (tonal as their contact calls in red-capped mangabeys vs. repeated noisy pulses as their alarm calls in guenons). Owren and Rendall (2001), suggested a “structure-affective processing” relationship: (1) call types used by callers to directly influence the affect of listeners would be characterized by peculiar acoustic features well-designed for capturing their attention (e.g., sharp onset, high-amplitude noisiness, repeated energy pulses) but not suitable for conveying individual distinctiveness; (2) call types used to indirectly influence the affect of listeners would give clear cues to caller identity (e.g., tonal harmonically-rich calls) that listeners would associate, through a conditioning process, with past positive or negative interactions with the caller. Accordingly, we found that identity coding was the strongest in the tonal contact calls regardless of the species, and that female mangabey tonal threat calls were more individually distinctive than their noisy alarm calls, whereas in guenons the noisy threat calls were the least individually distinctive. Owren and Rendall (2001) notably illustrated their “structure-affective processing” hypothesis with calls produced by baboons and macaques, contact and threat calls having indirect affective effects, distress and alarm calls having direct affective effects. Interestingly, we found a similar pattern in red-capped mangabeys whose social organization is based on relatively frequent peaceful and agonistic interactions and on a strong hierarchy like most baboons and macaques (Rowell, 1988; Dolado and Beltran, 2012). In contrast, guenons' social organization is based on rare physical interactions and on a discrete hierarchy (Gautier-Hion and Gautier, 1978; Rowell, 1988; Lemasson et al., 2006), and they displayed a different pattern here, with threat calls having an acoustic structure more likely to have direct affective effects on listeners. Consequently, we suggest that the call's relevance to the species' social organization (i.e., its role in mediating interactions that are crucial according to the species-specific social needs) accounts for its acoustic structure and its associated level of individual distinctiveness.

Taken together, these results suggest that the species-specific social needs have exerted a selective pressure on call structure, favouring acoustic variability (e.g., individual distinctiveness or context-related acoustic variability) in some particular calls involved in critical aspects of intra-group social functioning. Also, the theory of an influence of the call social function on its level of acoustic variability can be extended to an influence on the coevolution between its acoustic structure and the associated communicative capacities.

### Influence of social structure on vocal repertoire structuring and usage

In accordance with the “vocal grooming” hypothesis (Dunbar, 1998; Griebel and Oller, 2008), we found that female red-capped mangabeys called more than twice as much as female guenons. Also, in accordance with the “socially-driven repertoire complexity” hypothesis (Blumstein and Armitage, 1997; McComb and Semple, 2005; Freeberg, 2006; Freeberg and Harvey, 2008; Knotková et al., 2009; Gustison et al., 2012), we found that female mangabeys displayed the largest and most diverse vocal repertoire in terms of structural composition (greatest number of unit types and assembling patterns, highest diversity index: *DI* = 0.67), Campbell's monkeys showed an intermediate pattern (*DI* = 0.52), and De Brazza's monkeys produced the smallest and simplest repertoire (smallest number of unit types and assembling patterns, lowest diversity index: *DI* = 0.33). As red-capped mangabeys live in large multi-male multi-female groups, Campbell's monkeys in harem groups, and De Brazza's monkeys in small family units, our results suggest that the species' social structure account for the shaping of its vocal repertoire. While those evolutionary hypotheses have been, to date, tested separately in different taxa (e.g., primates: Dunbar, 1998, 2012; McComb and Semple, 2005; Gustison et al., 2012; rodents: Blumstein and Armitage, 1997; Knotková et al., 2009; Pollard and Blumstein, 2012; cetaceans: May-Collado et al., 2007; birds: Freeberg, 2006; Freeberg and Harvey, 2008; Krams et al., 2012), our study highlights, by using a multi-level approach (sound unit—assembling patterns—repertoire) of vocal variability in terms of both production and usage, that those hypotheses are complementary (Freeberg et al., 2012).

### From single sound utterance to more complex vocal combination

It is interesting to look more thoroughly at the way differential levels of vocal complexity are achieved across the three species. Parallel to the increase in social complexity between our three species, we found an increase in the number of unit types, in the number of assembling patterns, as well as in the number of “combined” patterns (i.e., concatenation of units of different types). One possibility of “creating” acoustic variability is to have non-fixed acoustic parameters (e.g., red-capped mangabey threat calls), but this has some limit in non-human primates as the control they have over their vocal apparatus is restricted (Lemasson, 2011). Another possibility of “creating” acoustic variability is to use syntactic-like sound combinations. The perceptual salience of those sound combinations remains to be tested for our subjects, nevertheless a growing number of recent studies provided evidence that those higher-level acoustic structures can be meaningful in a proto-syntactic-like way. Guenon males use suffixation (Campbell's monkeys: Ouattara et al., 2009a) and call sequences (putty-nosed monkeys: Arnold and Zuberbühler, 2008; Campbell's monkeys: Ouattara et al., 2009b) to increase the number of context-specific alarm messages delivered. Even in apes, it has been reported that white-handed gibbons concatenate the same set of notes into different songs (Clarke et al., 2006), chimpanzees combine calls (Crockford and Boesch, 2005), and bonobos (*Pan paniscus*) combine calls into sequences (Clay and Zuberbühler, 2011) in context-specific ways.

The ability of monkeys and apes to combine sound units or calls into more complex structures parallels, in some respects, the combinatorial ability pushed to extremes in human languages. The three closely-related forest species we studied here all used this possibility of syntactic-like sound combinations, but red-capped mangabeys who live in the most complex social groups are the ones who made the most of it. Also, combinatorial ability could have been enhanced in species whose social needs require individuals to increase the number of messages that they can deliver.

### Social complexity and the evolution of communicative abilities

Whereas broad inter-species comparisons based on a review of the existing literature suggest some patterns (e.g., repertoire size correlates with group size and time spent grooming in non-human primates: McComb and Semple, 2005), detailed comparative studies on a restricted number of closely-related species allow to take a closer look at how sociality has driven the evolution of communication (Freeberg et al., 2012). In a recent study, vocal repertoire size was compared between two species living in large social groups: geladas who aggregate into a complex multi-level society composed of small reproductive units within which one male forms long-term bonds with several females, and chacma baboons who live in a single-level multi-male multi-female society where cross-sex relationships consist mainly of temporary consortships (Gustison et al., 2012). The authors identified a number of homologous call types common to both species, but they found that geladas displayed a larger vocal repertoire than chacma baboons. The non-homologous (derived) call types specific to geladas function in cross-sex bonding, and they were produced primarily by males who are responsible for the maintenance of those long-term social bonds. Two interesting conclusions emerge from this study. First, social bonding turns out to be a key factor, more critical than the size of the reproductive unit, in the emergence of socially-driven vocal complexity in geladas. Second, the influence of social factors on vocal complexity appears to be more sizeable in males, as a result of their social role within the gelada society.

In our case, we focused on females whose role in mediating intra-group social relationships is predominant in the three species we studied. Therefore, we expected the influence of social factors on vocal variability to be most remarkable in this sex class. Overall, we found that female mangabeys, who are involved in a more complex social network than female guenons, display higher degrees of vocal complexity at several levels (acoustic variability, repertoire size, calling rate). Nevertheless, we do not rule out the possibility of socially-driven vocal complexity in males. It is notable that sex differences in vocal repertoire, though sizeable in the three studied species, are more striking in guenons (Campbell's monkeys: Gautier, 1988; Lemasson and Hausberger, 2011; De Brazza's monkeys: Bouchet et al., 2012b) than in red-capped mangabeys (Bouchet et al., 2010). Thus, relatively speaking, male mangabeys tend to equal the level of vocal complexity displayed by females (e.g., calling rate, vocal repertoire size). However, it is impossible here to discriminate between the effects of group size (larger in mangabeys than in guenons) vs. degree of involvement within the social network (male mangabeys are more socially active than male guenons). In the future, comparative studies on a small number of carefully chosen species might help uncover the relative influence of distinct aspects of sociality (e.g., group size, social network complexity, strength of social bonds) in the evolution of communicative abilities, as well as the extent to which it affects individuals unevenly according to their role within the social group.

So far, we have focused our discussion on comparative studies which, like ours, highlighted a parallel between social and vocal complexity in line with the theory of a social-vocal coevolution of communicative abilities. Interestingly, a study on gestural complexity in three species of macaques (*Macaca* sp.) showed a relationship between the complexity of social organization and the size of the gestural repertoire as well as the level of variability in communicative patterns (Maestripieri, 2007). More recently, a study on facial mobility in 12 non-human primate species revealed a correlation between group size and the variety of facial movements a species can produce (Dobson, 2009). Another study on chemical complexity of glandular secretions in eight species of brown lemurs (*Eulemur* sp.) that differ in their social system provided evidence of a correlation between social complexity and olfactory complexity (Del Barco-Trillo et al., 2012). Lastly, a study on the auditory system of 20 non-human primate species revealed a correlation between social complexity and enhanced hearing sensitivity (Ramsier et al., 2012). This set of comparative studies suggests that the theory of a social-vocal coevolution of communicative abilities might also apply to other communicative modalities at both the production and perception levels (Freeberg et al., 2012).

Our study points out the advantage of addressing multiple facets of communicative complexity in a small number of closely-related but socially distinct species. It opens new perspectives for comparative research on the evolution of communication in animal species. The existing literature suggests that socially-driven vocal complexity is widespread in the animal kingdom (reviewed in Freeberg et al., 2012). Targeted detailed comparative studies would help uncover whether or not similar social pressures (e.g., group size, social bonding) have affected identical aspects of communication (e.g., calling rate, repertoire size), and led to comparable levels of vocal complexity in disparate taxonomic groups. This approach applied, more specifically, to representatives of the primate lineage would be of great interest to advance our understanding of the selective pressures that led to the emergence of a communicative system as complex as human language.

### Conflict of interest statement

The authors declare that the research was conducted in the absence of any commercial or financial relationships that could be construed as a potential conflict of interest.
